# DNA Hypermethylation at the Invasive Front of Oral Squamous Cell Carcinoma Confers Poorly Differentiated Characteristics and Promotes Migration of Cancer Cells

**DOI:** 10.3390/diagnostics15192477

**Published:** 2025-09-27

**Authors:** Li-Po Wang, Chien-Ya Li, Yu-Hsueh Wu, Meng-Yen Chen, Yi-Ping Hsieh, Tze-Ta Huang, Tse-Ming Hong, Yuh-Ling Chen

**Affiliations:** 1 Institute of Oral Medicine, College of Medicine, National Cheng Kung University, Tainan 70101, Taiwan; lipowang0203@gmail.com (L.-P.W.); cianya1124@gmail.com (C.-Y.L.); yuhsueh1107@hotmail.com (Y.-H.W.); ccdc0002.tw@gmail.com (M.-Y.C.); tzetahuang@gmail.com (T.-T.H.); 2 Institute of Basic Medical Sciences, College of Medicine, National Cheng Kung University, Tainan 70101, Taiwan; pin1000822@gmail.com; 3 Institute of Clinical Medicine, College of Medicine, National Cheng Kung University, Tainan 70101, Taiwan; htmjimmy@gmail.com

**Keywords:** OSCC, epigenetic regulation, DNA methylation, 5-methylcytosine, differentiation, migration, EMT

## Abstract

**Background/Objectives:** Oral squamous cell carcinoma (OSCC) is a common and aggressive oral cancer with high recurrence and mortality rates, largely due to late diagnosis and metastasis. Epigenetic regulation, particularly aberrant DNA methylation, plays a critical role in cancer progression. Altered methylation patterns disrupt cancer-related gene regulation. Our previous study found that oral cancer patients exhibit increased synthesis of S-adenosyl-L-methionine, a key methyl donor for cytosine methylation. Therefore, the aim of this study was to explore the relationship between global DNA methylation and OSCC progression and to evaluate the impact of DNA methylation heterogeneity on oral cancer cells. **Methods:** Immunohistochemistry (IHC) and immunofluorescence (IF) staining were used to examine 5-methylcytosine (5-mC) expression in OSCC clinical specimens and oral cancer cells. The DNA methyltransferase inhibitor 5-Aza-dC was used to assess the effects of DNA methylation on cell function and gene expression. RNA sequencing was used to identify key differentially expressed genes affected by 5-Aza-dC treatment. Cell migration was assessed using a wound closure assay. Protein and gene expression were analyzed using Western blotting and quantitative PCR. **Results:** An inverse relationship was found between 5-mC levels and cancer differentiation—poorly differentiated OSCC exhibited higher 5-mC levels. Additionally, higher 5-mC staining was observed at the invasion front of oral cancer tissues. In OSCC cells, 5-mC content correlated with migration ability. Furthermore, conditioned medium from cancer-associated fibroblasts enhanced both methylation levels and migration of OSCC cells. Treatment with 5-Aza-dC significantly increased epithelial differentiation, reduced epithelial-to-mesenchymal transition and cell adhesion-related genes, and inhibited OSCC cell migration. **Conclusions:** The findings highlight the critical role of DNA hypermethylation in OSCC progression, particularly in regulating differentiation, migration, and EMT. The interplay between the tumor microenvironment and epigenetic modifications underscores the complexity of OSCC biology and opens avenues for innovative therapeutic strategies.

## 1. Introduction

Oral cancer refers to malignant tumors that develop in the oral cavity. In recent years, it has been among the top eight cancers in the United States and holds an even higher ranking in Asia [1]. Around 90% of oral cancers are squamous cell carcinoma (OSCC), which has been the most common type of oral cancer for the past two decades [2]. Recent studies have highlighted aberrant epigenetic regulation as a critical factor in oral cancer progression. Epigenetic mechanisms, which regulate gene expression without altering the DNA sequence, include DNA methylation, perturbations in histone post-translational modification patterns, alterations in chromatin remodeling, and deregulation of non-coding RNA expressions. Among these, DNA methylation is the most well-studied epigenetic mechanism [3]. This process involves the covalent modification of cytosine residues through the addition of a methyl group to the 5-carbon of the cytosine ring, forming 5-methylcytosine (5-mC). DNA methylation primarily occurs at cytosine-phosphate-guanine (CpG) dinucleotides, which are often concentrated in genomic regions known as CpG islands. Approximately half of all mammalian genes contain CpG islands near their promoters [4]. Changes in DNA methylation at these promoter regions regulate the activity of oncogenes and tumor suppressor genes [5].

In recent years, DNA hypermethylation has been widely recognized as a molecular marker for cancer. Hypermethylation of tumor suppressor gene promoter regions has been observed in nearly all cancer types including OSCC [6,7,8]. In recent years, our research on the impact of salivary microbiota on oral cancer has identified the increased synthesis of S-adenosyl-L-methionine, a key methyl donor for cytosine methylation, as the most significant change in OSCC patients [9]. Additionally, the balance between hypermethylation and hypomethylation is crucial, as disruptions can suppress or activate normal cellular activities in various genes [10]. Studies have also explored the role of abnormal global genomic methylation in carcinogenesis. For instance, hypermethylation has been observed in the early stages of cancer, including pancreatic cancer precursor lesions, as well as in oral leukoplakia and cancers of the gingivobuccal complex [11,12]. However, some evidence suggests that cancer cells predominantly undergo hypomethylation, leading to genomic instability and the reactivation of silenced genes [13,14].

Distinct levels of DNA methylation have been observed in specific tissue regions where malignant metastasis occurs in head and neck squamous cell carcinoma (HNSCC) [15,16]. Recent studies have also highlighted extensive genetic and non-genetic variations in tumor tissues, including heterogeneity in DNA methylation across different geographical regions of the tumor or at various stages of tumor progression [17]. Such heterogeneity in DNA methylation may drive phenotypic diversity, complicating cancer treatment. Understanding these variations offers valuable insights into tumor evolution and cancer biology, paving the way for more effective therapeutic strategies.

## 2. Materials and Methods

### 2.1. Clinical Samples and Patient Characteristics

A total of 132 formalin-fixed, paraffin-embedded OSCC tissue samples were obtained from the human biobank of National Cheng Kung University Hospital. This study received ethical approval from the Institutional Review Board (IRB) of National Cheng Kung University Hospital, Taiwan, on 21 January 2020 (IRB number: B-ER-108-424). All experiments involving clinical specimens were conducted in accordance with the guidelines of this IRB. Informed consent was waived for the use of the specimens from the human biobank of National Cheng Kung University Hospital.

### 2.2. Cell Lines and Culture

We cultivated OECM1 and OC2 cells in RPMI-1640 medium (Gibco, Billings, MT, USA), and HSC3 cells in Dulbecco’s modified Eagle’s medium (DMEM, Gibco). All the culture media were supplemented with 10% fetal bovine serum (Gibco) and 1× antibiotic-antimycotic solution (GeneDireX Inc., Taoyuan City, Taiwan) in a humidified incubator at 37 °C with 5% CO_2_.

### 2.3. Immunohistochemical Analysis and Scoring

**Figure 1:**
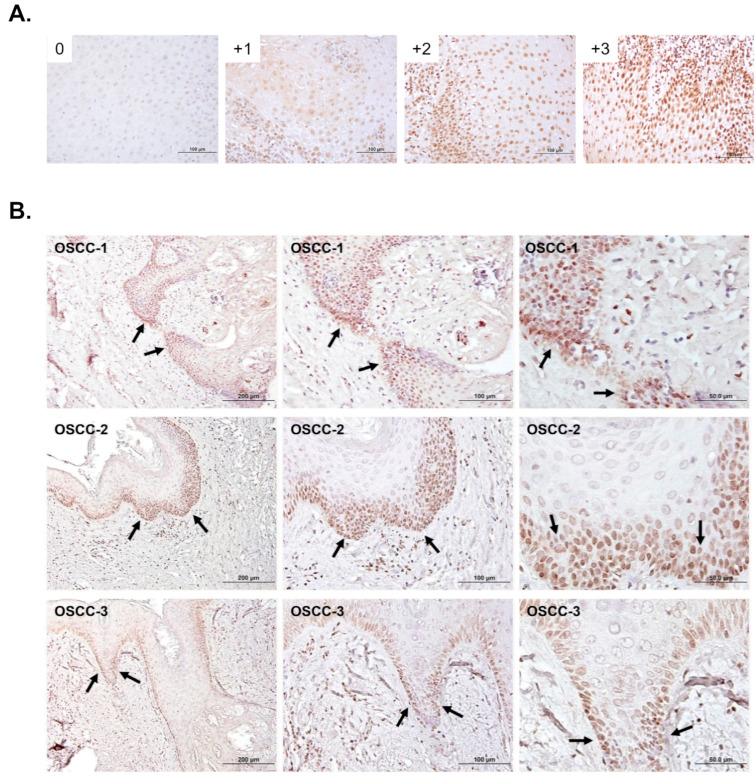
Immunohistochemical staining for 5-mC levels in OSCC specimens. (**A**) Immunoreactivity intensities in the nucleus (brown) were classified as low (scores 0–1) or high (scores 2–3) expression of 5-mC in OSCC. Scale bars: 100 μm. (**B**) Levels of 5-mC were elevated at the tumor invasive fronts of the lesion. Scale bars: 200, 100 and 50 μm. Arrows indicate the invasive fronts.

The immunohistochemistry procedure was carried out according to a previously published paper [18]. Primary antibody of 5-mC (MABE146, MilliporeSigma, Burlington, MA, USA) was diluted at a 1:500 dilution. The slides were examined under a light microscope and evaluated by an oral cancer pathologist (Dr. Yu-Hsueh Wu) and two researchers, who were blinded to the patient’s clinical information. We defined the expression of 5-mC in the nucleus of cancer cells as positive expression and classified it according to the staining intensity as weak (0–1) and strong (2–3). (Figure 1A). On the other hand, 5-mC expression at the invasion front was also assessed (Figure 1B).

### 2.4. In Vitro Wound-Healing Assay

OECM1, OC2, and HSC3 cells were placed into a culture insert (Ibidi Culture-Inserts, Gräfelfing, Germany) at a density of 3 × 10^4^ cells per well. Inserts were removed and washed with PBS after the cells were attached, then poured onto fresh medium to allow the cells to crawl for an appropriate period. Photographs of the wound gap were taken at 40× magnification at the beginning and end of the cell crawling period. Finally, calculated the area of cells migrated.

### 2.5. Genomic DNA Isolation and DNA Methylation Quantification

Genomic DNA (gDNA) was isolated using the Genomic DNA Mini Kit (Geneaid, New Taipei City, Taiwan) according to the manufacturer’s instructions. Genomic DNA was quantified for 5-mC using the Global DNA Methylation Assay Kit (5-mC, Colorimetric, Abcam, Cambridge, UK) and analyzed according to the manufacturer’s instructions.

### 2.6. Drug Treatment and CAF-Conditioned Medium

Isolation and culture of cancer-associated fibroblasts (CAFs) were performed according to a previous report [19]. CAF-conditioned medium was collected after culturing CAFs in DMEM medium without FBS for 24 h. OSCC cells were treated with DNA methyltransferase (DNMT) inhibitor 5-Aza-2′-deoxycytidine (5-Aza-dC) at a concentration of 1.25 µM for a total of 3 days and replaced with fresh medium containing 5-Aza-dC on day 2. OSCC cells were treated with 50% of CAF-conditioned medium for two days before 5-mC and cell migration analysis were performed.

### 2.7. RNA-Sequencing Analysis

RNA sequencing of 5-Aza-dC-treated OC2 cells was performed referring to previous studies [20]. Differential gene expression (DEG) analysis was conducted and identified using edgeR. Gene Ontology (GO) enrichment and Kyoto Encyclopedia of Genes and Genomes (KEGG) pathway analysis of DEGs were carried out using the R package 3.8.1 *p* < 0.01 was chosen as the cutoff criteria. Gene Set Enrichment Analysis (GSEA) was also performed using the ‘clusterProfiler’ package in R, and all visualization was handled in R using the ggplot2 graphics package. Significant enrichment pathways were defined by FDR < 0.25 and *p* < 0.05.

### 2.8. Statistical Analysis

All statistical analyses were performed using GraphPad Prism, V8.0 (GraphPad Software, Inc., San Diego, CA, USA). All values calculated using Student’s *t*-test are presented as the mean ± standard deviation (SD) from three independent experiments. Differences were considered significant at a *p*-value of <0.05.

## 3. Results

### 3.1. The Association of 5-mC Levels with Clinicopathological Characteristics of OSCC Specimens

To investigate the relationship between 5-mC levels and the clinicopathological parameters of oral cancer patients, samples from 110 patients were analyzed using immunohistochemistry. Antibody staining revealed 5-mC localized in the nuclei of cells. Two independent investigators scored the intensity of 5-mC staining into two categories: poor expression (scores 0–1) and strong expression (scores 2–3) (Figure 1A). Our analysis revealed an inverse correlation between 5-mC levels and differentiation grade, with less differentiated tissues exhibiting higher levels of 5-mC (Table 1). Furthermore, 5-mC was found to be highly expressed in cells at the leading edge of tumor invasion (Figure 1B).

### 3.2. Oral Cancer Cells with High 5-mC Levels Exhibit High Migration Ability

**Figure 2:**
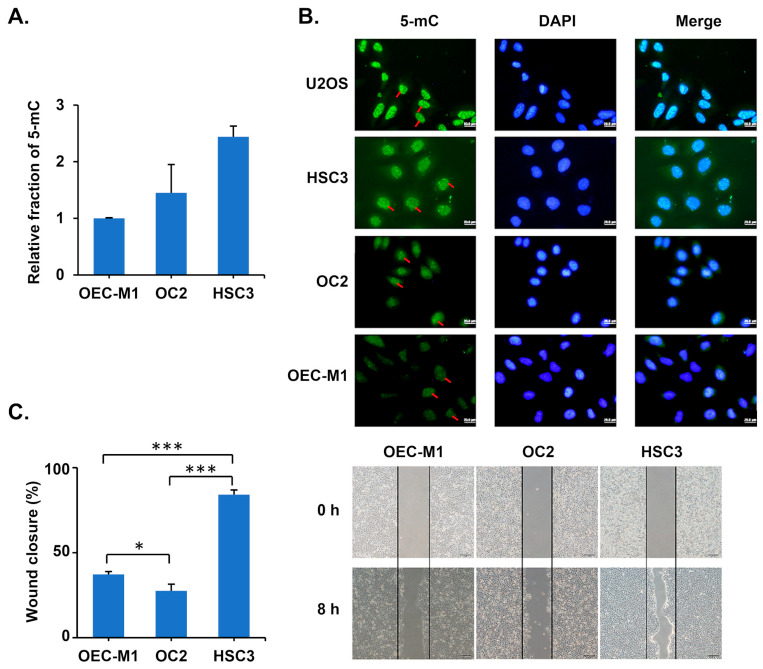
Oral cancer cell lines with high 5-mC content exhibit relatively high migration ability. (**A**) The levels of 5-mC in OECM1, OC2 and HSC3 cells were quantified using a Global DNA Methylation Assay Kit. (**B**) Immunofluorescence assay of 5-mC in oral cancer cell lines and U2OS cells. Nuclei were stained with DAPI, and red arrows indicate 5-mC signals. U2OS cells (human bone osteosarcoma epithelial cells) served as the positive control. Scale bars: 20 μm. (**C**) Left: Migration ability of oral cancer cells was assessed using a wound closure assay, with ImageJ 1.54h software used to measure the closed area. Data are presented as mean ± SD. * *p* < 0.05, *** *p* < 0.001, two-tailed Student’s *t*-test. Right: Representative images from the wound healing assay. Scale bars: 100 μm.

Given that 5-mC levels are elevated at the tumor invasion front, we hypothesized that high 5-mC levels in oral cancer cells may contribute to cancer invasion. To test this, we utilized an enzyme-based Global DNA Methylation Assay kit to measure the proportion of 5-mC in three oral cancer cell lines. The results indicated that OECM1 had the lowest 5-mC levels, followed by OC2, while HSC3 exhibited the highest 5-mC content (Figure 2A). Similarly, immunofluorescence analysis confirmed that nuclear 5-mC staining intensity was strongest in HSC3 cells, moderate in OC2, and lowest in OECM1 (Figure 2B). Furthermore, cell migration analysis using a wound closure assay revealed that HSC3 cells displayed the highest migration rate, while OC2 and OECM1 exhibited significantly lower migration rates (Figure 2C). Our previous studies have demonstrated that the invasion ability of these three OSCC cell lines is HSC3 > OC2 > OEC-M1 [21]. These results suggest that DNA methylation levels follow a similar trend in the migration and invasion abilities of OSCC cells.

### 3.3. Cancer-Associated Fibroblasts Increase 5-mC Levels in Oral Cancer Cells

**Figure 3:**
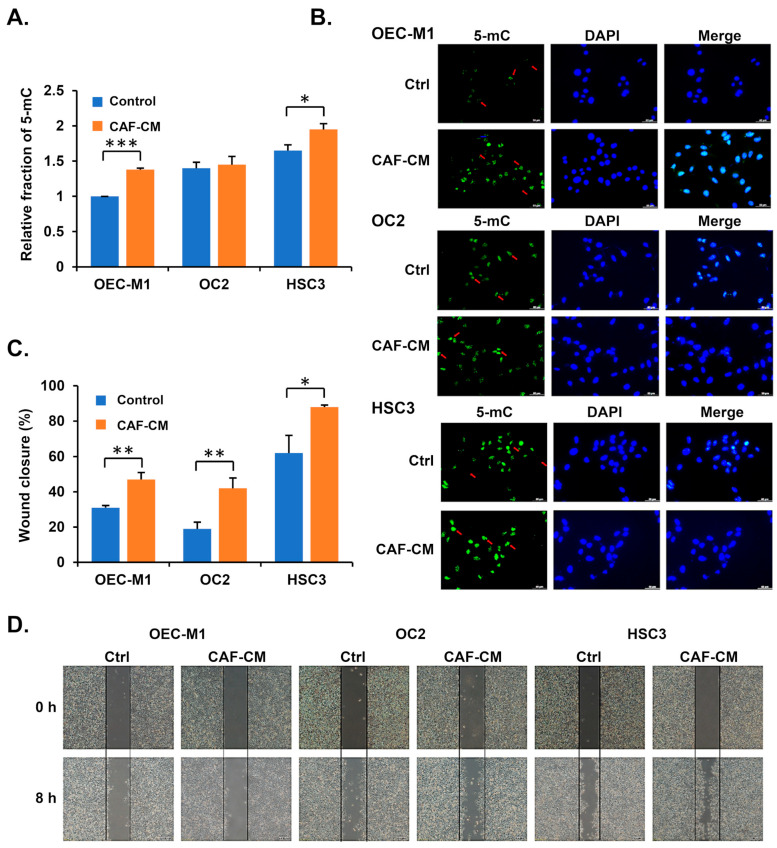
CAF-conditioned medium increases 5-mC levels and enhances the migration ability of OSCC cells. (**A**) The levels of 5-mC in OECM1, OC2 and HSC3 cell lines treated with CAF-conditioned medium (CAF-CM) for 2 days were quantified using the Global DNA Methylation Kit. Relative 5-mC levels were normalized to those of the OECM1 control group. (**B**) Immunofluorescence assay of 5-mC levels in HSC3, OC2, and OECM1 cells treated with CAF-CM for 2 days. Nuclei were stained with DAPI, and red arrows indicate 5-mC signals. Scale bars: 50 μm. (**C**) Migrated area was measured and quantified using ImageJ. Data are presented as mean ± SD. * *p* < 0.05, ** *p* < 0.01, *** *p* < 0.001, two-tailed Student’s *t*-test. (**D**) Representative images of wound healing assays at specific time points for OECM1, OC2, and HSC3 cells treated with CAF-CM. Scale bars: 100 μm.

The tumor invasion front represents the interface where cancer cells infiltrate surrounding tissues. We hypothesized that cancer-associated fibroblasts (CAFs) in the tumor microenvironment might influence the 5-mC levels of cancer cells at the leading edge of invasion. To test this, oral cancer cells were treated with the conditioned medium from two different oral cancer patients-derived CAFs. Both a global methylation detection kit and immunofluorescence analysis revealed that CAF-conditioned medium significantly increased 5-mC levels in OSCC cells (Figure 3A,B and Figure S1A). Additionally, OSCC cells treated with CAF-conditioned medium exhibited a marked increase in migratory ability (Figure 3C,D and Figure S1B). These findings suggest that fibroblasts in the tumor microenvironment may enhance 5-mC levels in OSCC cells, promoting a more aggressive cancer phenotype.

### 3.4. Decreasing 5-mC Reduces the Migration Ability of OSCC Cells

**Figure 4:**
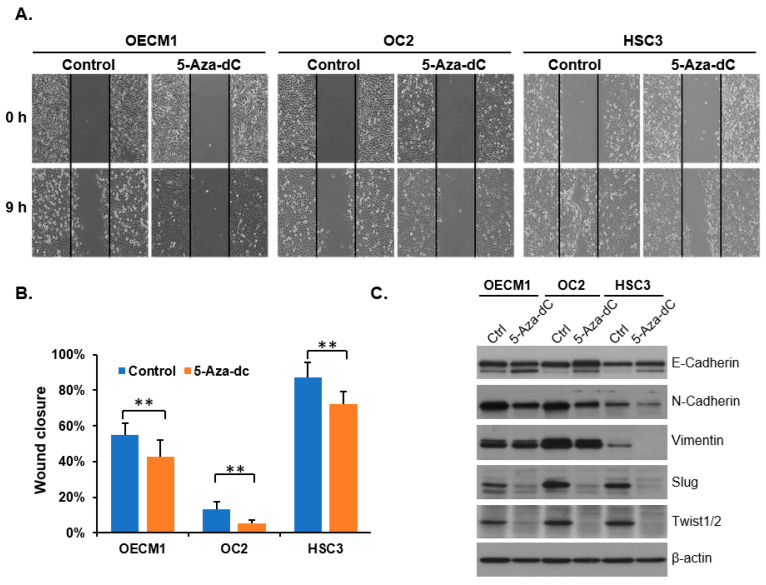
5-Aza-2-deoxycytidine treatment reduces the migration ability of OSCC cells. (**A**) Representative images of wound healing assays at specific time points in OECM1, OC2, and HSC3 cells treated with 5-Aza-2-deoxycytidine (5-Aza-dC) are shown. Scale bars: 100 μm. (**B**) The migrated areas were measured and quantified using ImageJ. Results are presented as mean ± SD. ** *p* < 0.01, two-tailed Student’s *t*-test. (**C**) Western blotting analysis of EMT markers. OSCC cells were treated with 1.25 μM 5-Aza-dC for 3 days and then subjected to Western blot analysis to examine EMT markers. DMSO-treated cells served as a control.

To investigate whether reducing the level of 5-mC affects cancer cell migration, we treated the cells with the DNA methylation inhibitor 5-Aza-2-deoxycytidine (5-Aza-dC) and analyzed their wound closure rate and epithelial–mesenchymal transition (EMT) properties. DNA methylation levels in OSCC cells were reduced after 72 h of 5-Aza-dC treatment (Supplementary Figure S2). The results demonstrated that 5-Aza-dC significantly inhibited the migration of oral cancer cells (Figure 4A,B). Western blot analysis revealed that 5-Aza-dC altered the expression of EMT-related proteins, with an increase in the epithelial marker E-Cadherin and a decrease in the mesenchymal markers N-Cadherin, Vimentin, Slug, and Twist (Figure 4C and Figure S3).

### 3.5. Reducing 5-mC Affects Gene Expression Related to Cell Differentiation and Migration

**Figure 5:**
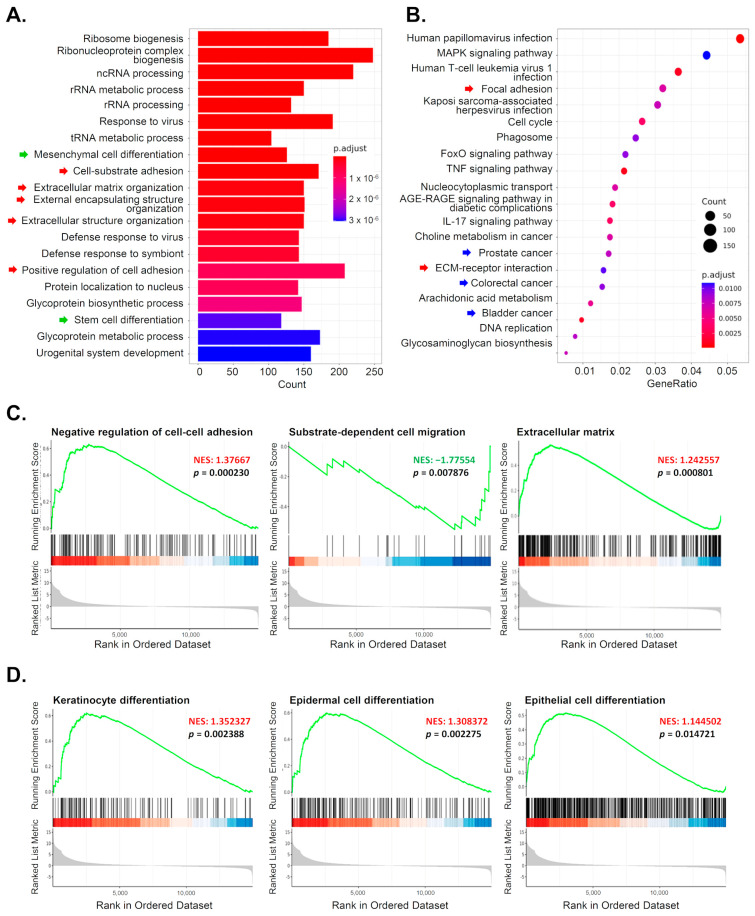
RNA-seq analysis reveals altered gene sets related to cell differentiation and migration in 5-Aza-dC-treated OSCC cells. OC2 cells were treated with DMSO or 5-Aza-dC for 72 h, followed by RNA extraction and RNA sequencing. (**A**) Gene Ontology (GO) enrichment analysis was performed on the differentially expressed genes. Biological processes related to “cell differentiation” and “cell adhesion” are highlighted by green and red arrows, respectively. (**B**) The top 20 enriched pathways identified in the KEGG database are shown. Pathways related to “focal adhesion” and “cancers” are marked by red and blue arrows, respectively. (**C**,**D**) Gene Set Enrichment Analysis (GSEA) of the RNA-seq data showed significant alterations in gene expression profiles. Genes associated with cell–cell and cell–substrate interactions are shown in (**C**), while genes involved in keratinocyte, epidermal cell, and epithelial cell differentiation are presented in (**D**).

To further understand how changes in global methylation levels affect gene expression in OSCC cells, we conducted RNA sequencing. After 72 h of treatment with 5-Aza-2-deoxycytidine (5-Aza-dC) or the control (DMSO), RNA from OC2 cells was sequenced. First, Gene Ontology (GO) enrichment analysis was performed on the differentially expressed genes (DEGs). In addition to processes related to RNA and ribosome biogenesis, biological processes (BP) associated with “cell differentiation” and “cell adhesion” were significantly altered by 5-Aza-dC treatment (Figure 5A). The top 20 enriched pathways in the KEGG database also included pathways related to “cell adhesion” (red arrow) and “cancer” (blue arrow) (Figure 5B).

Furthermore, Gene Set Enrichment Analysis (GSEA) of all genes from the RNA sequencing data revealed that the expression profiles in 5-Aza-dC-treated cells were enriched in genes associated with the “negative regulation of cell–cell adhesion,” while genes related to “substrate-dependent cell migration” were downregulated (Figure 5C). The GSEA results also showed that genes linked to “cell differentiation” in keratinocytes, epidermal cells, and epithelial cells were significantly enriched in 5-Aza-dC-treated cells (Figure 5D).

**Figure 6:**
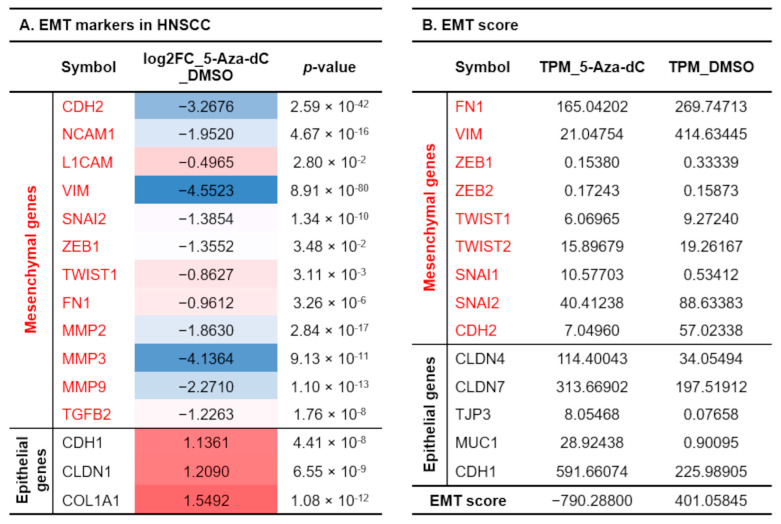
5-Aza-dC-treated cells show an epithelial phenotype. (**A**). A heatmap displays the differences in gene expression between 5-Aza-dC-treated and control cells for EMT-related genes in head and neck squamous cell carcinoma. Gene expression levels in 5-Aza-dC-treated cells were compared with those in the control group, and the log2FC values of gene expression were calculated. Red indicates increased log2FC gene expression in 5-Aza-dC-treated cells relative to the control group, while blue indicates decreased expression. (**B**). EMT score was defined by the sum of the expression of mesenchymal marker genes minus the expression of epithelial genes for each group. The higher EMT score connotes cell is approximately mesenchymal type. On the contrary, the lower EMT score connotes cell is approximately epithelial type.

Supplementary Table S1 lists genes significantly altered in 5-Aza-dC-treated cells, including those associated with EMT [22], cell–substrate adhesion [23], and epithelial differentiation [24]. Notably, inhibiting DNA methylation increased epithelial markers such as E-cadherin and claudin 1 in oral cancer cells, while reducing the expression of most mesenchymal markers like N-cadherin (*CDH2*), vimentin (*VIM*), metalloprotease 3 (*MMP3*), ZEB1 (*ZEB1*), and Twist1 (*TWIST1*) (Figure 6A). EMT score analysis has been used to predict the status of EMT in cancer cells [25]. An EMT score for each cell line was calculated based on analysis of RNA-sequencing data. We found that the EMT score of cells treated with 5-Aza-dC decreased from 401 to −790, indicating a significant epithelial phenotype transition (Figure 6B). Moreover, the expression of most genes related to cell–substrate adhesion, such as integrins, fibronectin, and MMPs, was also significantly reduced in 5-Aza-dC-treated cells. In summary, DNA hypermethylation in oral cancer cells inhibits the expression of epithelial gene sets, making the cells tend to have more invasive mesenchymal characteristics, while also increasing the expression of cell–matrix adhesion-related genes, driving the cells to become more malignant.

A recent study using single-cell analysis has revealed marker genes for different stages of keratinocyte differentiation [24]. RNA sequencing analysis revealed that 5-Aza-dC treatment decreased the expression of most basal keratinocyte markers, while significantly increasing the expression of spinous cell markers and granular keratinocyte markers, such as *KRT1*, *DEFB1*, *IVL*, *ZNF750*, and *CALML5* (Table 2). Taken together, the RNA sequencing results support the conclusion that inhibiting global DNA methylation reduces migration and affects cell differentiation in oral cancer cells.

## 4. Discussion

DNA methylation is a fundamental epigenetic modification that plays a crucial role in regulating gene expression and maintaining genome stability. Research analyzing DNA methylation patterns in OSCC has demonstrated distinct differences between normal, dysplastic, and cancerous tissues. For instance, aberrant methylation of promoter CpG islands has been observed across oral precancerous lesions and OSCC genomes, indicating that these epigenetic changes occur early in the disease process and may serve as predictive markers for cancer development [26]. Furthermore, studies have shown that the methylation status of specific genes can vary among different subsites of HNSCC, including the oral cavity, pharynx, and larynx, suggesting that epigenetic alterations may be influenced by the tumor’s anatomical location [27]. Overall, global DNA methylation studies provide valuable insights into the epigenetic landscape of oral cancer, highlighting the importance of DNA methylation alterations in the initiation and progression of OSCC.

We found that 5-mC was highly expressed in OSCC cells at the tumor invasion front, and oral cancer cells with elevated 5-mC levels showed enhanced migration. This spatial pattern links DNA methylation to invasiveness and highlights the role of epigenetic regulation in tumor progression. Regional variations in epigenetic states have been observed in various cancers, such as prostate [28], lung [29], and brain cancers [30] and can function in influencing tumor progression. RNA sequencing confirmed that inhibiting DNA methylation reduces the expression of cell–matrix interaction-related genes (e.g., integrins, fibronectin, MMP) and EMT status, thereby limiting the migration and invasion of OSCC. EMT-related genes, such as E-cadherin (*CDH1*), are often regulated by DNA methylation [31]. Hypermethylation of the *CDH1* promoter region is frequently reported in OSCC [32]. Downregulation of E-cadherin disrupts cell–cell adhesion, reduces tissue integrity, and promotes invasiveness and metastasis in OSCC. 5-Aza-dC treatment has been shown to induce MET progression in trophoblasts [33]. Transcriptome analysis highlights the potential of epigenetic therapies to reverse malignancy and improve prognosis. Targeting DNA methylation, such as with DNMT inhibitors or 5-Aza-dC, is a promising strategy to reduce OSCC invasion and metastasis [34]. DNMT inhibitors induce genomic hypomethylation; however, the mechanisms by which hypomethylation lead to the downregulation of genes like vimentin (*VIM*) and integrins (*ING*) remain unclear and warrant further investigation.

The tumor microenvironment, specifically stromal fibroblasts or CAFs, appears to influence 5-mC levels in OSCC cells. CAFs, a key component of the tumor microenvironment, significantly impact cancer progression through various mechanisms. They secrete a range of cytokines and growth factors that regulate the epigenetic landscape of tumor cells, thereby promoting cancer metastasis and enhancing cancer cell stemness [19,35,36]. CAF-conditioned medium has been shown to increase 5-mC levels in cancer cells, supporting the hypothesis that the tumor microenvironment actively contributes to promoting a more aggressive phenotype. Current literature highlights that epigenetic regulation of fibroblasts can shift their original anti-cancer barrier function into one that supports the malignant transformation of cancer cells [36]. However, there is limited discussion regarding whether the cytokines and growth factors secreted by CAFs influence the expression of DNMTs or other enzymes involved in maintaining the DNA methylation balance in cancer cells [37]. Consequently, the detailed mechanisms underlying this process remain to be further explored.

This study identified an inverse correlation between 5-mC levels and tumor differentiation grade. Less differentiated tissues exhibited higher levels of 5-mC, suggesting that DNA methylation may play a role in driving tumor cell dedifferentiation. Specifically, DNA methylation significantly influences cancer differentiation grades by regulating key genes involved in cell identity, maturation, and proliferation [38]. Previous studies have shown that treating transformed human keratinocytes with 5-Aza-dC promotes their differentiation, along with upregulation of the epidermal differentiation complex gene *IVL* and various keratin genes [39,40]. Furthermore, DNA methylation dynamics during epidermal differentiation align with changes in the expression of epigenetic factors responsible for these modifications [40]. In adult stem cells, differentiation is associated with a general decrease in the methylation of lineage-specific regulatory elements [41]. Collectively, these findings demonstrate the intricate functional complexity of DNA methylation in epidermal homeostasis and underscore the need for further investigation.

Bacteria induce DNA methylation at CpG sites in the host in two ways. One is to indirectly induce abnormal DNA methylation through inflammatory responses, and the other is to directly induce DNA methylation. For example, *Helicobacter pylori* infection and the upregulation of DNMT are caused by the infiltration of lymphocytes and macrophages, which in turn promote the secretion of interleukin (IL)-1β and nitric oxide by gastric epithelial cells [42]. *Escherichia coli* can directly upregulate DNMT activity and expression in human urothelial cells, leading to methylation of the cyclin-dependent kinase inhibitor 2A (CDKN2A) gene to downregulate CDKN2A expression, allowing pathogens to persist in the body [43]. Oral microbes induce changes in host cell DNA methylation, thereby promoting their own survival and persistence. These microbes or their products also directly or indirectly regulate the expression or function of DNMTs, leading to changes in host gene methylation patterns, thereby affecting host health or disease progression [44]. Oral pathogens like *Porphyromonas gingivalis* and *Fusobacterium nucleatum* have been shown to downregulate DNMT1 in host cells, influencing inflammatory gene expression and leading to periodontal disease [45]. In cancer, microbes-induced aberrant DNA methylation might silence tumor suppressor genes, contributing to malignancy. Our previous analysis of the salivary microbiome showed that the OSCC-associated microbiome has a greater potential for S-adenosyl-L-methionine (SAM) synthesis, especially in OSCC patients with oral submucous fibrosis (OSF) [9]. However, the results of this study showed that the levels of DNA methylation in tumor specimens from OSF-OSCC patients were no different from that in OSCC patients (Table 1). SAM serves as a universal methyl group donor in various biochemical reactions, including DNA, RNA, and protein methylation. It plays a crucial role in maintaining cellular homeostasis, regulating gene expression, and managing autophagy. SAM also participates in the synthesis of polyamines and plays a role in redox homeostasis [46]. Therefore, whether SAM influences the methylation of other types of biomolecules or the synthesis of polyamines, thereby regulating cancer progression, remains an important topic for future research.

## 5. Conclusions

The findings underscore the critical role of 5-mC in OSCC progression, particularly in regulating differentiation, migration, and EMT. The interplay between the tumor microenvironment and epigenetic modifications highlights the complexity of OSCC biology and opens avenues for innovative therapeutic strategies. Targeting DNA methylation with drugs could be promise in reducing OSCC aggressiveness and enhancing treatment efficacy. Future studies should aim to elucidate the precise molecular pathways regulated by 5-mC and assess the clinical potential of DNA methylation inhibitors in OSCC treatment.

## Figures and Tables

**Table 1:** Correlation between 5-methylcytosine levels and the clinicopathological variables in OSCC patients.

Clinicopathological Parameters	Number of Patients	5-Methylcytosine Level		*p*-Value
		Low No = 52	High No = 58	Fisher’s Exact Test
Age (years)				
>50	61	31	30	0.4462
≤50	49	21	28	
Tumor stage				
T1–T2	85	38	47	0.367
T3–T4	25	14	11	
Differentiation ^#^				
Gx-G1	71	41	30	0.0081 **
≥G2	37	11	26	
N stage				
N (−)	81	40	41	0.8234
N (+)	26	12	14	
Oral submucous fibrosis				
Yes	52	23	29	0.5713
No	58	29	29	
Site of tumor				
Buccal mucosa	44	24	20	
Gingiva/Mouth floor/Lip	28	11	17	0.6223
Tongue/Others	37	17	20	
Gender				
Male	106	50	56	1
Female	4	2	2	

^#^ The histological grade of the tumor was classified as follows: Gx (grade of differentiation cannot be assessed), G1 (well differentiated), G2 (moderately differentiated). ** *p* < 0.01.

**Table 2:** 5-Aza-dC treatment significantly increased the expression of keratinocyte differentiation-related markers in OC2 cells.

Basal keratinocyte markers			Spinous cell markers			Granular keratinocyte markers		
Symbol	log2FC_5-Aza-dC _DMSO	*p*-Value	Symbol	log2FC_5-Aza-dC _DMSO	*p*-Value	Symbol	log2FC_5-Aza-dC _DMSO	*p*-Value
KRT5	−0.6006	3.42 × 10^−3^	KRT1	4.5040	1.52 × 10^−17^	IVL	1.7620	2.12 × 10^−5^
CDC20	−0.6351	2.17 × 10^−3^	CDH1	1.1361	4.41 × 10^−8^	ZNF750	3.3005	7.56 × 10^−33^
RRM2	−0.4995	1.56 × 10^−2^	DEFB1	7.8417	6.17 × 10^−43^	SPINK5	0.9962	1.08 × 10^−4^
HELLS	−0.4273	4.45 × 10^−2^	FXYD3	2.1689	2.56 × 10^−17^	CALML5	4.8699	1.28 × 10^−55^
UHRF1	−0.7751	2.41 × 10^−4^	CCND1	−0.8113	8.82 × 10^−5^			
COL17A1	−0.5664	6.05 × 10^−3^						
GJB2	0.9503	4.27 × 10^−6^						
KRT16	2.4534	1.82 × 10^−28^						
ASS1	3.0584	5.59 × 10^−24^						

## Data Availability

The datasets used and/or analyzed during the current study are available from the corresponding author on reasonable request.

## Supplementary Materials

The following supporting information can be downloaded at: https://www.mdpi.com/article/10.3390/diagnostics15192477/s1, Supplementary Table S1 [22,23,24]: List of differentially expressed genes related to ECM, integrin and epidermal cell differentiation.; Supplementary Figure S1. CAF2-conditioned medium increases 5-mC levels and enhances the migration ability of OSCC cells.; Supplementary Figure S2. All OSCC cells exhibited demethylation after 5-aza-dC treatment.; Supplementary Figure S3. Western blot analysis of EMT-related proteins.
