# Supplementary material for: DNA Hypermethylation at the Invasive Front of Oral Squamous Cell Carcinoma Confers Poorly Differentiated Characteristics and Promotes Migration of Cancer Cells
Source: Diagnostics (Basel). 2025 Sep 27;15(19):2477. doi: 10.3390/diagnostics15192477 (PMC12523504; doi:10.3390/diagnostics15192477)
Supplement: Supplementary file 1 [file diagnostics-15-02477-s001.zip › diagnostics-3870733-supplementary.pdf]

Supplementary Table 1. List of differentially expressed genes related to ECM, integrin and epidermal cell differentiation.

Symbol	Description	log2FC_5-Aza_DMSO	*p*-value
**Epithelial markers[1]**			
CDH1	cadherin 1; E-cadherin	1.1361	4.40641E-08
CLDN1	claudin 1	1.2090	6.54565E-09
COL1A1	collagen type I alpha 1 chain	1.5492	1.08301E-12
**Mesenchymal markers[1]**			
CDH2	cadherin 2; N-cadherin	-3.2676	2.58998E-42
NCAM1	neural cell adhesion molecule 1	-1.9520	4.67052E-16
L1CAM	L1 cell adhesion molecule	-0.4965	0.028439084
VIM	vimentin	-4.5523	8.91022E-80
SNAI1	snail family transcriptional repressor 1	4.0300	3.65388E-17
SNAI2	snail family transcriptional repressor 2, Slug	-1.3854	1.33796E-10
ZEB1	zinc finger E-box binding homeobox 1	-1.3552	0.034844927
TWIST1	twist family bHLH transcription factor 1	-0.8627	0.003112313
TGFB2	transforming growth factor beta 2	-1.2263	1.75535E-08
**Cell-substrate adhesion related genes[2]**			
ITGA1	integrin subunit alpha 1	-0.9477	6.86795E-05
ITGA2	integrin subunit alpha 2	-0.6924	0.000795745
ITGA3	integrin subunit alpha 3	-1.4051	1.61323E-11
ITGA6	integrin subunit alpha 6	-1.2924	5.14704E-10
ITGA7	integrin subunit alpha 7	1.4909	0.111029597
ITGAV	integrin subunit alpha V	-0.1965	0.343413762
ITGB1	integrin subunit beta 1	-0.5684	0.005649404
ITGB2	integrin subunit beta 2	1.1577	3.41321E-07
ITGB4	integrin subunit beta 4	-0.7742	0.000168202
ITGB5	integrin subunit beta 5	-1.2675	2.72305E-09
ITGB6	integrin subunit beta 6	-0.4215	0.045042117
ITGB7	integrin subunit beta 7	4.6733	1.49655E-16
ITGB8	integrin subunit beta 8	0.8383	0.000199937
FN1	fibronectin 1	-0.9612	3.26021E-06
MMP2	matrix metallopeptidase 2	-1.8630	2.83639E-17
MMP3	matrix metallopeptidase 3	-4.1364	9.12761E-11
MMP9	matrix metallopeptidase 9	-2.2710	1.10376E-13
**Basal keratinocyte markers[3]**			
KRT5	keratin 5	-0.6006	0.003423484
CDC20	cell division cycle 20	-0.6351	0.002173097
RRM2	ribonucleotide reductase regulatory subunit M2	-0.4995	0.01555072
HELLS	helicase, lymphoid specific	-0.4273	0.044522717
UHRF1	ubiquitin like with PHD and ring finger domains 1	-0.7751	0.000240694
COL17A1	collagen type XVII alpha 1 chain	-0.5664	0.006050222
KRT19	keratin 19	12.9740	1.3324E-203
GJB2	gap junction protein beta 2	0.9503	4.271E-06
KRT16	keratin 16	2.4534	1.82028E-28
ASS1	argininosuccinate synthase 1	3.0584	5.59388E-24
**Spinous cell markers[3]**			
KRT1	keratin 1	4.5040	1.52211E-17
CDH1	cadherin 1, E-cadherin	1.1361	4.40641E-08
DEFB1	defensin beta 1	7.8417	6.16515E-43
FXYD3	FXYD domain containing ion transport regulator 3	2.1689	2.56474E-17
CCND1	cyclin D1	-0.8113	8.81614E-05
**Granular keratinocyte markers[3]**			
IVL	involucrin	1.7620	2.1164E-05
ZNF750	zinc finger protein 750	3.3005	7.56165E-33
SPINK5	serine peptidase inhibitor Kazal type 5	0.9962	0.000107886
CALML5	calmodulin like 5	4.8699	1.28286E-55

[1] Pawlicka M, Gumbarewicz E, Błaszczak E, Stepulak A. Transcription Factors and Markers Related to Epithelial-Mesenchymal Transition and Their Role in Resistance to Therapies in Head and Neck Cancers. Cancers (Basel). 2024 Mar 29;16(7):1354. doi: 10.3390/cancers16071354.

[2] Janiszewska M, Primi MC, Izard T. Cell adhesion in cancer: Beyond the migration of single cells. J Biol Chem. 2020 Feb 21;295(8):2495-2505. doi: 10.1074/jbc.REV119.007759.

[3] Wang S, Drummond ML, Guerrero-Juarez CF, Tarapore E, MacLean AL, Stabell AR, Wu SC, Gutierrez G, That BT, Benavente CA, Nie Q, Atwood SX. Single cell transcriptomics of human epidermis identifies basal stem cell transition states. Nat Commun. 2020 Aug 25;11(1):4239. doi: 10.1038/s41467-020-18075-7.

Supplementary Fig. 1

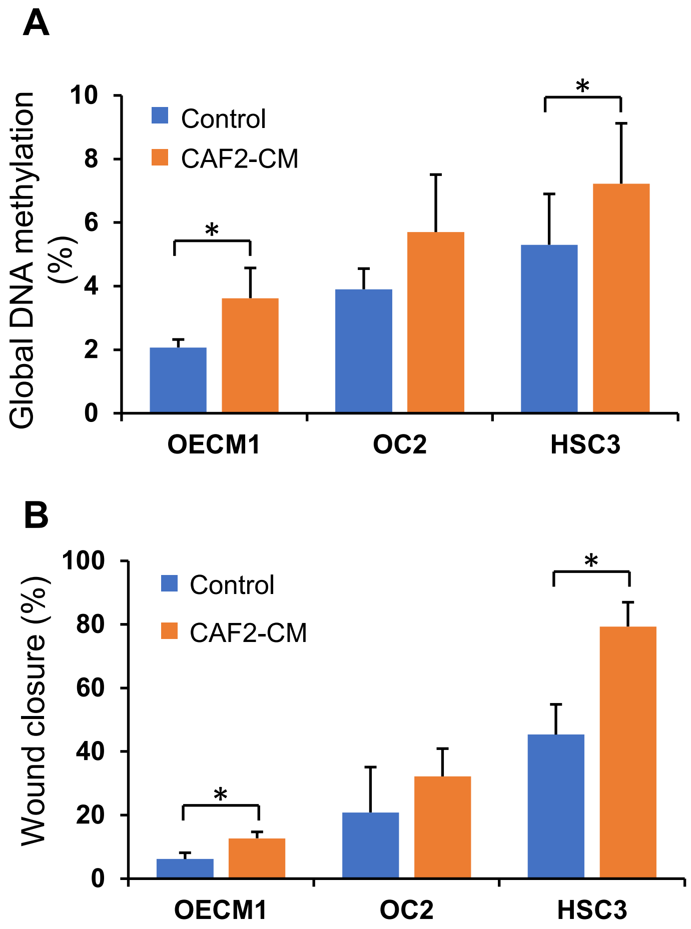

Supplementary Figure 1. CAF2-conditioned medium increases 5-mC levels and enhances the migration ability of OSCC cells.

(A) The levels of 5-mC in OECM1, OC2 and HSC3 cell lines treated with CAF2-conditioned medium (CAF2-CM) for 2 days were quantified using the Global DNA Methylation Kit. (B) Migrated area was measured and quantified using ImageJ. Data are presented as mean ± SD. *$P < 0.05$, two-tailed Student's t-test.

Supplementary Fig. 2

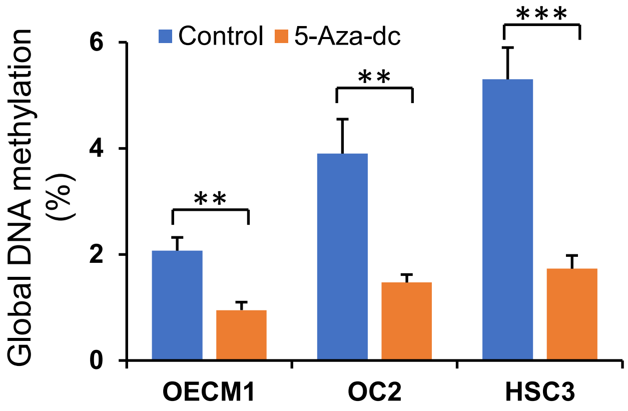

Supplementary Figure 2. All OSCC cells exhibited demethylation after 5-aza-dC treatment.

OSCC cells were treated with 1.25 μM 5-Aza-dC for 3 days, and global DNA methylation levels were quantified using a global DNA methylation detection kit. DMSO-treated cells served as a control. Results are presented as mean ± SD. $^{**}P < 0.01$, and $^{***}P < 0.001$, two-tailed Student's *t*-test.

Supplementary Fig. 3

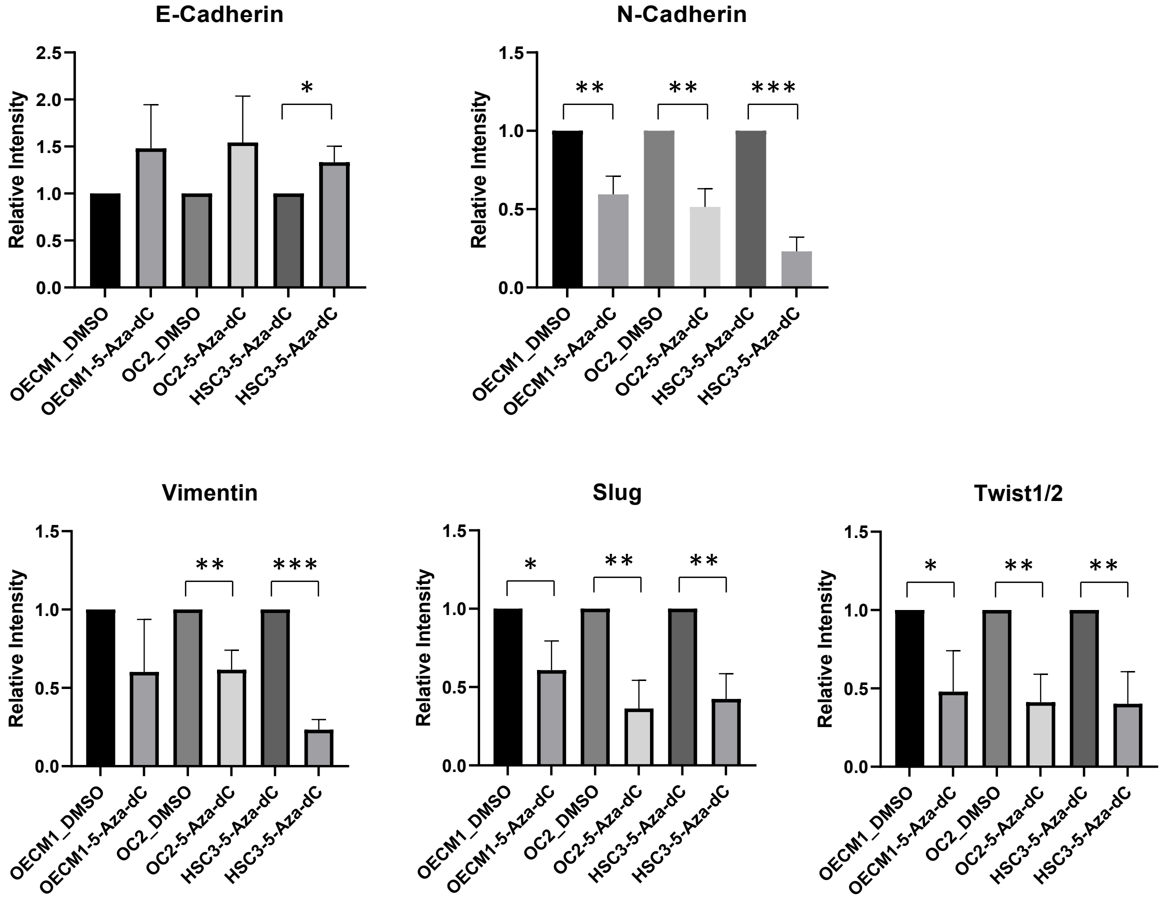

Supplementary Figure 1. Western blot analysis of EMT-related proteins

OSCC cells were treated with the 1.25μM 5-Aza-dC for 3 days, after which total proteins were extracted and subjected to Western blot analysis to assess EMT markers. Band intensities for each EMT marker were quantified using ImageJ from three independent experiments. *P < 0.05, **P < 0.01, and ***P < 0.001, two-tailed Student's *t*-test.
